# ARGLU1 in Glioma: A Novel Potential Regulator of Splicing, DNA Repair, and Therapeutic Resistance

**DOI:** 10.3390/cells15121124

**Published:** 2026-06-22

**Authors:** Xi Wu, Dongye Yi, Dongjun Tie, Mengqi Du, Meiying Wang, Zhuang Yu, Younian Xu

**Affiliations:** 1 Department of Anesthesiology, Union Hospital, Tongji Medical College, Huazhong University of Science and Technology, Wuhan 430022, China; wu2018whuh@163.com (X.W.); 15008616505@163.com (D.T.); mengqidu1326@163.com (M.D.); docwangmy@163.com (M.W.); 2 Institute of Anesthesia and Critical Care Medicine, Union Hospital, Tongji Medical College, Huazhong University of Science and Technology, Wuhan 430022, China; 3 Key Laboratory of Anesthesiology and Resuscitation, Huazhong University of Science and Technology, Ministry of Education, Wuhan 430022, China; 4 Department of Neurosurgery, Union Hospital, Tongji Medical College, Huazhong University of Science and Technology, Wuhan 430022, China; dongye_yi@hotmail.com; 5 Department of Anesthesiology, Shanghai General Hospital, Shanghai Jiao Tong University School of Medicine, Shanghai 200080, China

**Keywords:** ARGLU1, glioma, RNA splicing, DNA repair, therapeutic resistance

## Abstract

**Highlights:**

**What are the main findings?**

- ARGLU1 is a potential three-in-one nuclear hub that couples RNA polymerase II pausing, alternative splicing, and homologous recombination repair in glioma.
- *ARGLU1* may contribute to temozolomide and radiotherapy resistance by enhancing spliceosome activity and BRCA2/RAD51-mediated DNA repair, though direct evidence in glioma is still limited. Its expression level might predict PARP inhibitor response, but glioma-specific validation is lacking.

**What are the implications of the main findings?**

- The unique triple functionality of ARGLU1 makes it an attractive therapeutic target to overcome chemoresistance and radioresistance, yet its intrinsically disordered nature calls for innovative strategies such as PROTACs.
- *ARGLU1* expression and its splicing signatures hold promise as prognostic biomarkers and precision therapy guides, pending systematic validation in glioma-specific cohorts.

**Abstract:**

ARGLU1 (Arginine and Glutamate Rich1) is a newly identified nuclear protein with suggested multifunctional roles that may be implicated in the pathogenesis and therapeutic resistance of glioma, the most common primary malignant brain tumor. The high heterogeneity and treatment resistance of gliomas pose central challenges in clinical management. ARGLU1 has been implicated in maintaining genomic stability and may contribute to tumor progression by regulating RNA splicing and DNA damage repair pathways. This review systematically summarizes the structural and functional features of ARGLU1 and discusses its potential molecular mechanisms in glioma. These include its influence on the spliceosome assembly, alternative splicing events, and key DNA repair pathways such as homologous recombination (HR) and Fanconi anemia (FA). Furthermore, it discusses the hypothesis that ARGLU1 may enhance DNA repair capacity and thereby influence glioma resistance to temozolomide (TMZ) and radiotherapy. Targeting *ARGLU1* may offer a strategy to overcome this resistance. Finally, the review outlines current research limitations and future directions, aiming to provide a new theoretical foundation for the precision treatment of glioma.

## 1. Introduction

Glioma, particularly glioblastoma (GBM), represents the most aggressive malignancy of the central nervous system, with a median patient survival of less than 15 months [1,2]. Despite standard treatments (surgery, radiotherapy, and temozolomide [TMZ]), tumors often recur and become resistant [3]. To survive therapy, tumor cells remodel RNA splicing and DNA repair. Aberrant alternative splicing (AS) helps glioma grow by producing oncogenic proteins and increasing heterogeneity [4]. Meanwhile, faulty DNA damage repair (DDR) pathways also help glioma cells resist DNA-damaging agents like TMZ and radiation [5].

ARGLU1 is a recently identified nuclear protein with an initial implication in RNA processing. Subsequent evidence derived from non-glioma cellular models suggests that *ARGLU1* participates in spliceosome regulation and is linked to DNA repair pathways involving *BRCA2* and *RAD51* [6,7]. Collectively, these observations establish *ARGLU1* as a potential molecular link between post-transcriptional regulation and the maintenance of genomic stability. Analogous splicing regulators, such as U6 snRNA-associated Sm-like protein 2 (*LSM2*), drive glioma progression by altering splicing and DNA repair [8]. Whether *ARGLU1* operates via a comparable mechanism remains to be experimentally addressed. Another pathway relevant to DNA damage repair is the Fanconi anemia (FA) pathway, which repairs interstrand crosslinks. Its potential involvement in glioma therapy resistance is discussed in Section 5.5.

Currently, no published study has directly investigated *ARGLU1* expression levels, clinicopathological relevance, or prognostic significance specifically in glioma tissues. This represents a clear knowledge gap that necessitates prompt and dedicated investigation. Although direct evidence is lacking, a recent study identified an *ARGLU1*-*NTRK1* gene fusion in an *IDH*-mutant anaplastic astrocytoma, suggesting a possible genetic link between *ARGLU1* and glioma [9]. Elucidating ARGLU1 function in glioma is therefore essential for understanding drug resistance and developing novel therapeutic strategies. This review explores three core hypotheses: firstly, whether *ARGLU1* may promote glioma stem cell maintenance through specific splicing events; secondly, whether it may function as a homologous recombination accessory factor conferring resistance to TMZ and *PARP* inhibitors; and thirdly, whether *ARGLU1* could serve as a clinically applicable biomarker for precision therapy in glioma. This review is structured as a hypothesis-driven mechanistic synthesis rather than an exhaustive literature compilation. Each proposed function of ARGLU1 is critically evaluated, with explicit distinction drawn between experimentally validated findings and speculative models. Most functional data on ARGLU1 have been derived from non-glioma cell lines. Thus, the findings discussed here require validation in glioma-specific models.

## 2. Molecular Structure and Functional Basis of ARGLU1

### 2.1. Gene Locus and Protein Domains of ARGLU1

**Figure 1:**
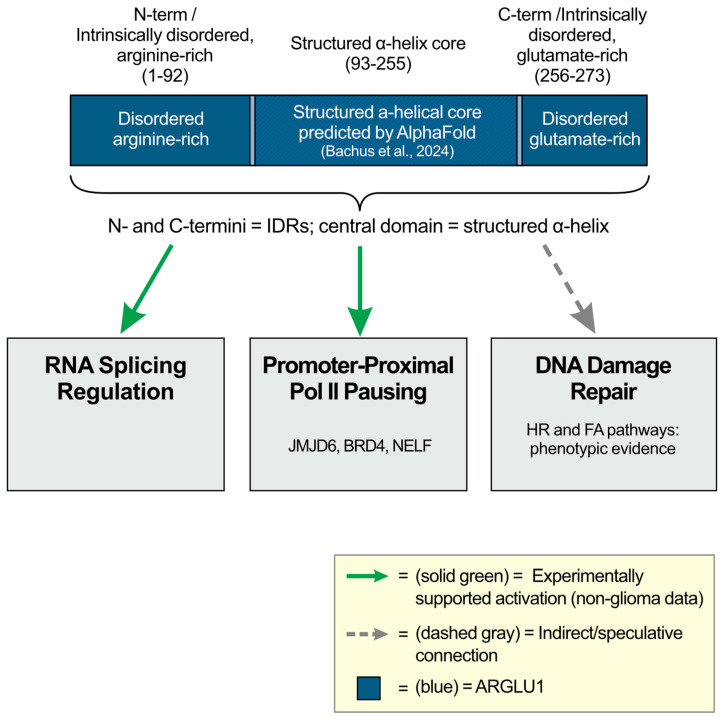
Segmented domain architecture and associated functional pathways of ARGLU1. ARGLU1 comprises three structurally distinct regions: an N-terminal intrinsically disordered region (residues 1–92, arginine-rich), a structured core α-helical domain (residues 93–255) as predicted by AlphaFold modeling [7], and a C-terminal intrinsically disordered region (residues 256–273, glutamate-rich). Three functional modules are displayed below: (1) RNA splicing regulation, (2) promoter-proximal Pol II pausing (with JMJD6, BRD4, NELF), and (3) DNA damage repair (HR and FA pathways: phenotypic evidence). Solid green arrows indicate experimentally supported activation (non-glioma data). Dashed gray arrows indicate indirect or speculative connections. The blue rectangle represents ARGLU1. Abbreviations: IDRs, intrinsically disordered regions; Pol II, RNA polymerase II; NELF, negative elongation factor; HR, Homologous recombination; FA, Fanconi anemia.

The structural properties of Arginine and Glutamate Rich 1 (ARGLU1) remain largely unexplored. Computational analyses, including AlphaFold modeling, predict that ARGLU1 is characterized by a structured central α-helical domain with intrinsically disordered regions (IDRs) at both the N-terminus and C-terminus [7]. This domain architecture suggests that ARGLU1 may exert its functions through unconventional mechanisms, potentially involving protein–protein interactions mediated by its disordered regions. The protein’s name derives from its amino acid composition, being rich in arginine and glutamate residues, which may contribute to its interactions with nucleic acids or other proteins [10]. Elucidating its precise three-dimensional structure and the functional implications of intrinsically disordered regions are critical areas for future investigations aimed at understanding ARGLU1’s roles in cellular regulation [7] (Figure 1).

### 2.2. Expression and Function of ARGLU1 in Normal Tissues

In normal cellular contexts, ARGLU1 participates in RNA splicing and transcriptional regulation [11]. Its role extends to the DNA damage response pathway, where it plays a part in promoting DNA damage repair, indicating a function in maintaining genomic stability [7]. In addition to its potential role in glioma, ARGLU1 has been implicated in breast cancer through its cooperation with MED1 in facilitating estrogen receptor-mediated transcription, and in gastric cancer as a potential therapeutic target [10,12]. Collectively, these findings point to evolutionarily conserved oncogenic functions of ARGLU1 across multiple solid tumor types. At the mechanistic level, ARGLU1 influences transcription by enhancing promoter-proximal RNA polymerase II pausing, a key regulatory step in gene expression [7]. This pausing effect is likely achieved by ARGLU1 inhibiting the interaction between JMJD6 and BRD4, two proteins involved in transcriptional elongation [7]. Thus, ARGLU1 functions as a nuclear factor that coordinates RNA processing fidelity with transcriptional dynamics under physiological conditions.

### 2.3. Interaction of ARGLU1 with the Spliceosome Complex

While ARGLU1 is recognized for its role in RNA splicing, the specific details of its interaction with the spliceosome complex have yet to be fully elucidated [11]. Its role as a splicing factor implies a direct or indirect association with the core spliceosomal machinery, although the precise binding partners and the underlying mechanisms remain to be characterized [10]. The intrinsically disordered regions of ARGLU1 may facilitate its interactions with multiple components of the splicing apparatus [7,11]. Furthermore, studies on viral infection have provided additional context. For example, ARGLU1 interacts with the adenovirus E1A protein, which manipulates host cell processes including splicing, highlighting the connection between ARGLU1 and RNA metabolism [13]. This viral interaction model thus serves as a useful tool to probe the native functions of ARGLU1 within the splicing regulatory network.

## 3. The Critical Challenge of Glioma and the Potential Role of ARGLU1

### 3.1. The Aggressive Nature and Therapeutic Hurdles of Glioblastoma

Glioblastoma (GBM) remains the most aggressive primary malignant brain tumor, with a median survival of less than 15 months despite standard multimodal therapy involving surgery, radiotherapy, and temozolomide (TMZ) [14,15]. High rates of tumor recurrence and the development of therapeutic resistance are major contributors to treatment failure, highlighting the urgent need to elucidate the underlying molecular mechanism [3]. Recent research has increasingly focused on how glioma cells survive therapy by remodeling RNA splicing and DNA repair, two processes now recognized as key drivers of resistance [16]. This adaptive reprogramming allows tumors to survive genotoxic insults, highlighting the urgency of identifying novel therapeutic targets.

### 3.2. ARGLU1: A Novel Node Connecting RNA Processing and Genome Stability

ARGLU1 is a recently identified nuclear protein initially implicated in RNA processing. However, current evidence from non-glioma models suggests its function extends beyond splicing regulation, positioning it as a potential contributor in glioma pathogenesis [7,11]. Intriguingly, ARGLU1 may participate in BRCA2/RAD51-associated repair processes, although direct interaction has not been demonstrated [17]. Nevertheless, existing support for direct ARGLU1 binding to BRCA2 or RAD51 relies primarily on predicted protein interaction networks and indirect functional studies [7]. Direct biochemical evidence from co-immunoprecipitation or pull-down assays remains limited, underscoring a key technical gap in the field. Nonetheless, the dual involvement of ARGLU1 in both post-transcriptional regulation and the maintenance of genomic stability positions it as a candidate molecular hub worth investigating. Investigating its precise role in glioma could offer novel insights into the integrated mechanisms of therapy resistance and provide a foundation for developing innovative treatment strategies [18].

A particularly informative model for elucidating ARGLU1’s involvement in DNA repair derives from studies of adenovirus infection. Koul et al. demonstrated that ARGLU1 acts as a restriction factor that suppresses the adenoviral replication cycle through direct binding to the viral E1A protein [13]. Importantly, this interaction diminishes DNA damage repair in bleomycin-treated cells, indicating that E1A binding compromises ARGLU1’s pro-repair activity [13]. This finding carries two significant implications for glioma biology. Firstly, it provides functional evidence that ARGLU1’s repair-promoting function can be modulated via protein interactions, suggesting that endogenous binding partners may similarly regulate ARGLU1 in cancer cells. Secondly, the E1A-ARGLU1 interaction reveals a potential synthetic lethal vulnerability. Disruption of ARGLU1’s repair function by E1A-mimetic peptides or small molecules could sensitize glioma cells to DNA-damaging therapies without affecting normal cells. Collectively, these viral insights provide a basis for designing ARGLU1-targeted therapeutic sensitization approaches.

### 3.3. ARGLU1 as a Transcriptional Regulator: Mechanisms and Functional Implications

Beyond its roles in splicing regulation and DNA repair coordination, ARGLU1 also directly modulates transcription. A thorough understanding of this aspect of ARGLU1 biology is essential for assessing its regulatory role in glioma cells.

#### 3.3.1. Enhancement of Promoter-Proximal RNA Polymerase II Pausing

ARGLU1 controls transcription primarily by promoting RNA polymerase II (Pol II) pausing near promoter regions [7]. ARGLU1 binds JMJD6, which interacts with the negative elongation factor (NELF) complex [7]. Through this interaction, ARGLU1 stabilizes NELF at paused Pol II complexes, increasing the pausing index 20 to 60 nucleotides downstream of transcription start sites [10]. Enhanced Pol II pausing acts as a rate-limiting step for gene activation, enabling cells to respond rapidly to environmental signals while avoiding premature transcriptional elongation [10].

#### 3.3.2. Modulation of Transcription Factor Recruitment

*ARGLU1* also regulates transcription by modulating the recruitment of sequence-specific transcription factors to target gene promoters. Experimental evidence indicates that *ARGLU1* increases the occupancy of *SP1* and *YY1* at specific promoters, thereby influencing downstream gene expression [12]. For example, *ARGLU1* promotes transcription of mismatch repair genes, including *MLH3* and *MSH2*, revealing a novel link between ARGLU1-dependent transcriptional control and the maintenance of genome integrity [12].

#### 3.3.3. Coordination with DNA Damage Signaling and Repair

*ARGLU1* promotes DNA damage repair in response to genotoxic stress [7]. This integration of transcription and DNA repair positions *ARGLU1* as a regulator of genotoxic defense, with potential implications for therapy resistance in glioma.

#### 3.3.4. Viral Interaction and Implications for DNA Repair

During adenovirus infection, ARGLU1 directly binds the viral E1A protein, an interaction that inhibits viral gene transcription and limits viral replication [13]. Notably, the E1A-ARGLU1 complex interferes with host DNA damage repair pathways, indicating that ARGLU1’s repair-associated functions can be hijacked by viral proteins [13]. This finding offers a useful experimental model for elucidating how ARGLU1 natively coordinates transcription with DNA repair and suggests possible synthetic lethal interactions for therapeutic targeting.

#### 3.3.5. Biological Significance in Cancer and Development

Dysregulated ARGLU1 expression has been reported in several malignancies [19]. Elevated ARGLU1 levels promote cancer cell proliferation and drive chemotherapy resistance, whereas ARGLU1 depletion suppresses tumor growth [10]. Beyond its roles in cancer, ARGLU1 may also be involved in developmental processes and cell differentiation by modulating transcriptional pausing and gene expression programs that govern cell fate decisions [11]. These observations position ARGLU1 as a versatile regulator at the crossroads of transcription, RNA processing, and DNA repair, highlighting its potential as a therapeutic target in glioma.

## 4. The Landscape of Alternative Splicing Dysregulation in Glioma

### 4.1. Prognostic Significance of Splicing Events and Molecular Subtypes

Systematic profiling of alternative splicing (AS) has revealed its prognostic impact in glioma. AS-based signatures from low-grade gliomas (LGG) and GBM data can stratify patient survival with high accuracy [20]. Additionally, AS signatures involving genes such as mutY DNA glycosylase (*MUTYH*) and catenin beta 1 (*CTNNB1*) refine classifications based on *IDH* and 1p/19q status [21]. For example, alternative splicing of neurofibromin 1 produces a less active isoform in over 80% of high-grade gliomas, activating RAS-MAPK signaling and correlating with poor survival [16]. Thus, AS is a clinically relevant contributor to glioma heterogeneity.

### 4.2. Splicing Factors and Regulatory Mechanisms Driving Oncogenesis

Dysregulation of AS in glioma is driven by alterations in splicing factors and RNA-binding proteins. Key regulators such as polypyrimidine tract-binding protein 1 (PTBP1), methyltransferase-like 3 (METTL3), and small nuclear ribonucleoprotein D2 polypeptide (SNRPD2) have been identified as central drivers of oncogenic splicing program [22]. For example, PTBP1 promotes intersectin 1 (*ITSN1*) exon inclusion, shifting the balance to the oncogenic ITSN1-S isoform and enhancing glioma growth and invasion [23]. METTL3 facilitates heterogeneous nuclear ribonucleoprotein H1 (HNRNPH1)-dependent splicing of *LINC00475*, producing a splice variant that promotes mitochondrial fission and glioma progression [24]. Furthermore, SNRPD2 is a dependency hub in aggressive gliomas. Its high expression correlates with poor prognosis and drives oncogenic splicing events [18]. A comparison of *ARGLU1* with these regulators is presented in the Discussion (Section 8). Unlike other splicing regulators such as RNA-binding motif protein 4, quaking homolog, and select members of the serine/arginine-rich splicing factor family, ARGLU1 is unusual in its combined involvement in transcriptional pausing regulation and DNA repair coordination. This potential triple-function property remains to be validated in glioma-specific models. Collectively, these factors represent actionable targets whose inhibition could disrupt pro-tumorigenic splicing networks.

### 4.3. Splicing Heterogeneity at Single-Cell Resolution and in the Tumor Microenvironment

Single-cell transcriptomics have revealed the intricate AS landscape that distinguishes glioma cell states and shapes the immune ecosystem. The most divergent splicing patterns occur between mesenchymal and neuronal-like glioma cells, involving key regulators such as transcription factor 12 (*TCF12*) and polypyrimidine tract-binding protein 2 (*PTBP2*) [25]. Inducing a specific *TCF12* exon inclusion shifts glioma cells toward a neuronal phenotype and increases radiosensitivity [25]. AS events in tumor-infiltrating immune cells also correlate with clinical outcomes. For instance, membrane spanning 4-domains A7 (*MS4A7*) splicing in macrophages affects anti-PD-1 response, indicating that AS modulates immunotherapy efficacy [25]. This single-cell perspective suggests that therapeutic strategies may require consideration of isoform-level regulation across the entire tumor ecosystem.

### 4.4. Alternative Splicing Targets with Therapeutic Potential in Glioma

It is important to note that, except for Bcl-extra (*BCLX*), none of the AS events below have been linked to *ARGLU1*. They serve as examples of targetable events in glioma for future investigation. Beyond known splicing regulators, specific AS events create actionable vulnerabilities in glioma. These include immunogenic neoepitopes, targetable surface proteoforms, and functional circRNAs. Together, they highlight the therapeutic potential of the splice isoform repertoire in glioma.

#### 4.4.1. *OY-TES-1* Splice Variant V5a as a Driver of Malignancy

The *OY-TES-1* gene encodes a cancer-testis antigen and produces four distinct splice variants in glioma through alternative splicing [26]. Among these, the V5a isoform shows the highest percent spliced-in (PSI) value and correlates significantly with poorer overall survival [27]. *OY-TES-1*-V5a expression was an independent risk factor for poor prognosis, correlating with tumor grade and *IDH1* mutation status [27]. Functional studies demonstrated that ectopic V5a expression enhances glioma cell proliferation, migration, and invasion while suppressing apoptosis [27]. Notably, this variant is barely expressed in normal brain, making it a promising target for splicing modulation or immunotherapy. Whether *ARGLU1* directly regulates this splicing event remains unknown.

#### 4.4.2. *RCAN1-4*: A Splicing-Derived Neoepitope for TCR-T Cell Therapy

The regulator of calcineurin 1 (*RCAN1*) gene produces an immunogenic splice variant known as *RCAN1-4*. In glioblastoma (GBM), C/EBPβ drives *RCAN1-4* isoform expression, which is high in mesenchymal tumors and glioma stem cells [28]. *RCAN1-4* generates a unique splice junction epitope spanning exons 4–5. This epitope is restricted to human leukocyte antigen A24 (HLA-A24) and activates T cells in both healthy donors and GBM patients [28]. Using this epitope, researchers isolated *RCAN1-4*-reactive TCRs and engineered T cells that killed *RCAN1-4*-positive GBM cells *in vitro* and *in vivo* [28]. Thus, splicing-derived neoantigens can be functionally relevant even in low-mutation cancers such as GBM. This finding offers a generalizable platform for mining tumor-specific splice variants across human cancers [29]. Direct regulation of *RCAN1-4* splicing by *ARGLU1* has not been reported.

#### 4.4.3. *BCLX* Splicing Modulation to Overcome Apoptotic Resistance

Among the AS events discussed here, *BCLX* is the only one directly linked to *ARGLU1* [30]. Through alternative splicing, the *BCLX* gene produces two opposing isoforms: the anti-apoptotic Bcl-xL (from the distal 5′ splice site) and the pro-apoptotic Bcl-xS (from the proximal 5′ splice site). In GBM cells, aberrant splicing of *BCLX* pre-mRNA favors Bcl-xL expression, leading to apoptotic resistance and worse outcomes [30]. Splice-switching oligonucleotides (SSOs) blocking the distal splice site shift the balance toward Bcl-xS, activating apoptosis and autophagy in GBM cells. Radiation alone increases the Bcl-xL/Bcl-xS ratio, which may foster resistance. Combining SSOs with radiation improves radiosensitivity in GBM models without toxicity to normal astrocytes [30]. Thus, this strategy offers a promising approach for re-sensitizing GBM cells to genotoxic therapies.

#### 4.4.4. *NRCAM* Microexon Skipping as a Targetable Surface Proteoform

In pediatric high-grade glioma (pHGG), microexon skipping is the most common splicing alteration [31]. The neuronal cell adhesion molecule (*NRCAM*) shows skipping of exons 5 and 19 in nearly all pHGG samples [31]. The Δex5Δex19 variant is essential for migration, invasion, and tumor growth [31]. It is surface-expressed, making it targetable by antibodies. A monoclonal antibody against Δex5Δex19 NRCAM facilitates T cell-mediated killing via an FcRI-based receptor [31]. This supports targeting surface splice variants for adoptive immunotherapy [32]. The connection between *ARGLU1* and *NRCAM* microexon skipping is speculative and requires experimental validation.

#### 4.4.5. Circular RNAs in Glioma Pathogenesis

Some circular RNAs (circRNAs) act as oncogenes (e.g., circular RNA *NT5E*) or tumor suppressors (e.g., circular RNA *FBXW7*, circular RNA *SHPRH*) in glioma [33,34,35,36]. CircRNAs show diagnostic potential [35,36]. However, circRNA-based therapies are still at an early stage, and none have been linked to ARGLU1 [37,38].

#### 4.4.6. Clinical Translation and Remaining Challenges

**Figure 2:**
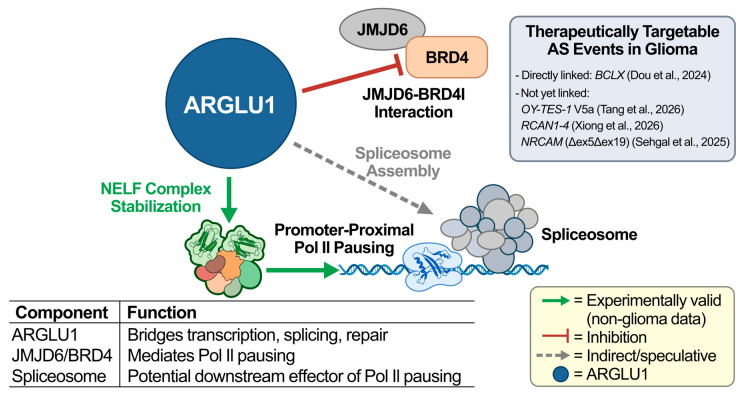
Schematic mechanism of ARGLU1-mediated co-transcriptional regulation. ARGLU1 (blue circle) disrupts the JMJD6-BRD4 interaction (red blunt arrow). This stabilizes NELF and enhances promoter-proximal Pol II pausing (solid green arrows). Through Pol II pausing, ARGLU1 may indirectly influence spliceosome assembly (dashed gray arrow). The right panel lists therapeutically targetable alternative splicing events: *BCLX* [30] is directly linked to *ARGLU1*; *OY-TES-1* V5a [27], *RCAN1-4* [28], and *NRCAM* (Δex5Δex19) [31] are not yet linked. The bottom table summarizes the functions of ARGLU1 and its partners. Solid green arrows: experimentally validated activation (non-glioma data). Red blunt arrow: inhibition. Dashed gray arrow: indirect/speculative connection. Blue: ARGLU1. Abbreviations: NELF, negative elongation factor; Pol II, RNA polymerase II; AS, Alternative splicing.

Splicing-targeted therapies face several hurdles in glioma. These include poor drug delivery across the blood–brain barrier, patient-to-patient variability in splicing, off-target toxicity, and the early stage of most studies. Despite these challenges, tumor-specific splice variants remain promising targets (Figure 2).

### 4.5. The Molecular Mechanism by Which ARGLU1 Regulates Alternative Splicing

ARGLU1 influences splicing by modulating the JMJD6-BRD4 interaction, which enhances Pol II pausing. Pausing is a key step linking transcription to alternative splicing [7]. No CLIP-seq or RNA-seq data are available to identify direct RNA targets of *ARGLU1*. Thus, its binding to specific pre-mRNAs remains unknown. Despite this, ARGLU1 promotes cancer cell growth and drug resistance, and its knockdown suppresses growth [7,12]. ARGLU1 also restricts adenovirus replication by enhancing Pol II pausing [13]. As noted in the Introduction, these findings come from non-glioma cells and need confirmation in glioma models.

### 4.6. ARGLU1-Mediated Splicing Variants and the Maintenance of Glioma Stem Cell Characteristics

Glioma stem cell (GSC) maintenance depends on the regulation of alternative splicing, a process potentially involving ARGLU1. Hypoxia suppresses *MBNL1*, triggering an adult-to-fetal splicing switch that promotes GSC self-renewal and tumor initiation [39]. Thus, splicing factor activity is linked to stem cell phenotypes. ARGLU1 plays emerging roles in DNA repair and transcription, pathways essential for GSC maintenance and therapy resistance [7]. Additionally, splicing isoform switches in *CERS5* and *MPZL1* regulate sphingolipid metabolism and SHP2 signaling, pathways associated with stem cell biology [22]. Given its involvement in Pol II pausing and splicing, *ARGLU1* may generate splice variants that sustain GSC phenotypes, thereby driving tumor progression and therapy resistance.

## 5. DNA Damage Repair Mechanisms and Their Contribution to Therapy Resistance

### 5.1. Key DNA Repair Pathways in Glioma Cell Survival

DNA repair pathways are fundamental for glioma cell survival after standard genotoxic therapies. TMZ induces O6-methylguanine as its primary cytotoxic lesion, which is directly repaired by O6-methylguanine-DNA methyltransferase (MGMT), making *MGMT* promoter methylation a key predictive biomarker for TMZ response [3]. In addition, base excision repair (BER) processes N7-methylguanine and N3-methyladenine lesions, whereas homologous recombination (HR) and non-homologous end joining (NHEJ) are critical for repairing DNA double-strand break (DSB) caused by radiotherapy [17]. Mismatch repair (MMR) can also occur, leading to a hypermutated phenotype that may alter therapeutic sensitivity [25]. The efficiency of these interconnected pathways directly influences whether glioma cells succumb to therapy or develop resistance, making them a central focus for combination treatment strategies [40].

### 5.2. DNA Repair as a Mechanism of Radioresistance and Chemoresistance

Enhanced DNA repair capacity is a well-established mechanism of resistance to radiotherapy and chemoresistance in glioma. For example, activation of the DNA-dependent protein kinase (DNA-PK) complex in the NHEJ pathway and proteins like RAD51 in the HR pathway enables tumor cells to efficiently repair radiation-induced damage [41]. TMZ resistance can also be driven by upregulated HR activity or MMR deficiency, both of which prevent the conversion of TMZ-induced lesions into lethal double-strand breaks [40]. Studies show that inhibiting specific repair pathways, such as using PARP inhibitors to impair BER in MMR-deficient cells, can resensitize resistant glioma cells to TMZ [42]. Additionally, factors like Yes-associated protein (YAP) can promote radio-resistance by enhancing DNA damage repair through fibroblast growth factor 2 transcription and MAPK-ERK pathway activation [43]. Targeting these repair adaptations is therefore a promising strategy to overcome resistance.

### 5.3. DNA Repair-Related Gene Signatures for Prognosis Prediction

The expression patterns of DNA damage repair (DDR) genes have significant prognostic value in glioma. Integrative analyses have established multi-gene signatures based on DDR gene expression that stratify patients into high- and low-risk groups with distinct survival outcomes [44]. These signatures often include genes involved in various repair pathways, such as *WEE1*, *RAD51*, and *PARP* family members, with high-risk scores correlating with advanced tumor grade, wild-type *IDH* status, and poor prognosis [45]. Nomograms incorporating these molecular signatures with clinical parameters such as age and Karnofsky performance score have been constructed, offering improved individualized prognostic prediction beyond clinical factors alone [46]. This highlights the translational potential of DDR pathways, not only as therapeutic targets but also as a basis for biomarker-driven patient stratification.

### 5.4. The Synergistic Mechanism of ARGLU1 and Homologous Recombination Repair

Based on evidence from non-glioma cell lines, *ARGLU1* appears to synergize with the homologous recombination (HR) repair pathway, although this has not yet been tested in glioma cells [7]. Mechanistically, ARGLU1 may facilitate HR by promoting Pol II pausing, thereby regulating HR-related gene expression. The *E1A*-*ARGLU1* interaction reduces DNA repair, indicating that *ARGLU1*’s repair function is modulated by binding partners [13]. Thus, ARGLU1 may act as an HR co-factor, enabling efficient DNA repair in cancer cells.

### 5.5. The Potential Association Between ARGLU1 and the Sensitivity of PARP Inhibitors

**Figure 3:**
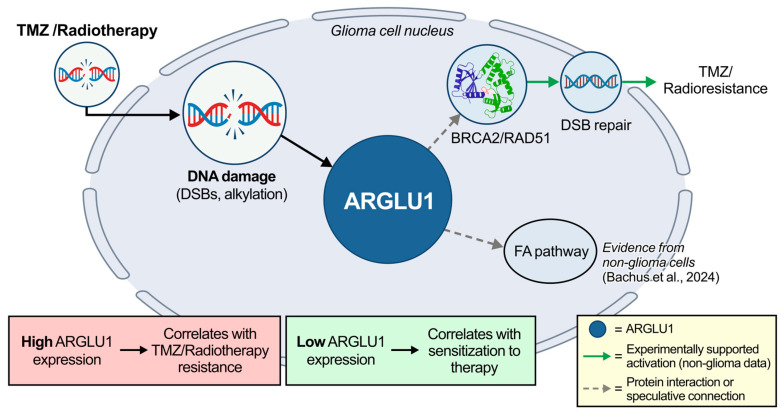
Proposed model of ARGLU1 in DNA repair and therapy resistance. TMZ or radiation induces DNA damage. ARGLU1 may be involved in HR repair through BRCA2/RAD51 (dashed gray arrow from ARGLU1 to BRCA2/RAD51, speculative) leading to DSB repair and therapy resistance (solid green arrows, experimentally validated from non-glioma data). ARGLU1 may also be involved in the FA pathway (dashed gray arrows, speculative; evidence from non-glioma cells [7]). High ARGLU1 expression correlates with resistance; low expression correlates with sensitization. Solid green arrows: experimentally validated activation (non-glioma data). Dashed gray arrows: indirect/speculative connection. Blue: ARGLU1. Abbreviations: DSB, double-strand break; FA, Fanconi anemia; HR, homologous recombination; Pol II, RNA polymerase II; TMZ, temozolomide.

The potential association between *ARGLU1* and PARP inhibitor sensitivity remains hypothetical, as direct evidence in glioma is lacking. PARP inhibitors exploit synthetic lethality in HR-deficient tumors [47]. Based on non-glioma cell data, *ARGLU1* overexpression could theoretically reduce PARP inhibitor efficacy [7]. Conversely, *ARGLU1* knockdown might enhance sensitivity [7]. The *E1A*-*ARGLU1* interaction also reduces DNA repair, raising the possibility that targeting *ARGLU1* could increase PARP inhibitor susceptibility [13]. However, these are hypothetical mechanisms derived from non-glioma systems, and rigorous glioma-specific validation is required before clinical application. The DNA repair pathways involving *ARGLU1*, as illustrated in Figure 3, provide a mechanistic rationale for exploring PARP inhibitor sensitivity.

## 6. The Interplay Between Splicing Dysregulation and DNA Repair in Fostering Resistance

### 6.1. Splicing Alterations in DNA Repair Genes

A direct link between splicing dysregulation and therapy resistance comes from alternative splicing events occurring within genes encoding DNA repair proteins. Such events can produce protein isoforms with altered or dominant-negative functions that compromise repair fidelity or efficiency. *NTRK2* (TrkB) is primarily a neurotrophic receptor, yet the receptor’s signaling indirectly modulates DNA repair. A truncated, kinase-deficient splice variant called TrkB.T1 is common in gliomas [48]. It amplifies oncogenic pathways such as PI3K/Akt and STAT3, which promote a more aggressive phenotype [48]. Mendelian randomization studies have causally linked specific splice isoforms of genes such as centrosomal protein 192 and fas apoptotic inhibitory molecule to increased glioma risk [49]. These findings implicate alternative splicing as a direct mechanism in gliomagenesis and the modulation of DNA repair pathways. Consequently, the splicing machinery acts as an oncogenic driver by rewiring the DNA damage response network, thereby creating therapeutic vulnerabilities that may be exploitable.

### 6.2. Coordinated Regulation by RNA-Binding Proteins and Splicing Factors

The coordination between RNA processing and DNA repair is often mediated by specific RNA-binding proteins (RBPs) and splicing factors that have dual functions or physically interact with repair complexes. Proteins such as LSM2, initially identified as part of the spliceosomal machinery, have been shown to drive glioma progression by causing widespread splicing alterations that affect pathways central to DNA damage response and cell cycle regulation [8]. Knockdown of *LSM2* alters 2000 splicing events, indicating its broad regulatory function [8]. Furthermore, Myc-associated zinc finger protein has been identified as a key transcription factor in resistant NPC-like glioma stem cells, enhancing DNA repair and stemness and driving TMZ resistance and poor prognosis [50]. These proteins integrate splicing dysregulation with genome maintenance defects, thereby reinforcing the resistant phenotype.

### 6.3. DNA Repair and Therapeutic Resistance: Targeting Repair Pathways to Overcome Chemo- and Radio-Resistance

The response of gliomas to conventional genotoxic treatments, namely temozolomide (TMZ) and radiotherapy, depends primarily on the tumor’s intrinsic capacity for DNA lesion recognition and repair. Therefore, pharmacologically inhibiting DNA repair pathways has become a rational strategy to re-sensitize therapy-resistant glioma cells. Among the various approaches, two mechanistically distinct strategies have drawn considerable attention: PARP inhibition under mismatch repair (MMR)-deficient conditions and direct suppression of repair effectors alongside TMZ.

#### 6.3.1. PARP Inhibition in MMR-Deficient Glioblastoma

MMR system deficiencies, which normally correct base-base mismatches and insertion-deletion loops, occur frequently in glioblastoma after TMZ treatment. MMR-deficient GBM cells tolerate TMZ-induced O^6^ methylguanine lesions due to their inability to recognize mispairs, thus avoiding the abortive repair cycle that normally triggers cell death. In this context, PARP inhibitors (PARPi) have been shown to restore TMZ sensitivity [42,51,52]. Mechanistically, PARPi impairs base excision repair (BER), leading to single-strand break accumulation that collapses into lethal double-strand breaks during replication. Notably, this synthetic lethal interaction between PARPi and MMR deficiency operates independently of BER function, as demonstrated by sustained PARPi-mediated sensitization after BER enzyme inhibition [42]. Preclinical evidence indicates that PARPi combined with TMZ significantly inhibits the growth of MMR-deficient GBM xenografts, warranting clinical investigation of this combination in recurrent disease [53].

#### 6.3.2. Direct Inhibition of DNA Repair as a Sensitization Strategy

Beyond PARP, other DNA repair factors have been effectively targeted to enhance TMZ and radiation sensitivity. Disabling homologous recombination (HR) or non-homologous end joining (NHEJ) impairs accurate double-strand break repair, tipping the balance in favor of cell death [54]. As an example, combining TMZ with DNA-PK or ATM inhibitors exhibits synergistic killing in preclinical glioma models [40]. Elevated expression of HR factors such as RAD51 contributes to TMZ resistance, whereas *RAD51* knockdown restores drug sensitivity [55]. These findings indicate that targeting multiple DNA repair pathways may be an effective strategy to overcome therapy resistance.

#### 6.3.3. Clinical Implementation and Ongoing Obstacles

Despite robust preclinical evidence, the clinical application of DNA repair inhibitors in glioma faces several obstacles. One major challenge is the blood–brain barrier, which limits central nervous system exposure of numerous small-molecule inhibitors and therefore requires chemical optimization or novel delivery approaches. Patient selection remains a key consideration; biomarkers such as MMR and HR status can help identify individuals most likely to benefit. Potential toxicities, particularly bone marrow suppression and gastrointestinal effects, necessitate careful dose scheduling. Nevertheless, ongoing trials are evaluating PARP, ataxia telangiectasia and Rad3-related protein (ATR), and DNA-PK inhibitors in combination with TMZ or radiation, with results expected to guide future therapeutic strategies [56,57].

## 7. Therapeutic Strategies to Overcome Splicing and DNA Repair-Mediated Resistance

### 7.1. Targeting Splicing Regulators and the Spliceosome

Given the central role of splicing dysregulation, direct targeting of the spliceosome or key splicing factors is a promising therapeutic strategy. Preclinical studies have demonstrated that pharmacological inhibition of core spliceosome components or regulatory RBPs can suppress tumor growth and overcome resistance. For example, targeting *PTBP1*, a regulator of an oncogenic AS signature linked to neural developmental hierarchies, suppresses tumor growth in adult glioma models and promotes differentiation [22]. Similarly, targeting *SNRPD2*, a dependency hub in aggressive gliomas, could disrupt its associated oncogenic splicing repertoire [18]. These approaches aim to reverse pro-tumorigenic splicing programs, potentially re-sensitizing cells to conventional therapies. However, clinical application of spliceosome inhibitors is challenging because splicing is also essential in normal cells, necessitating strategies to achieve a therapeutic window.

### 7.2. Inhibiting DNA Repair Pathways as a Sensitization Strategy

Inhibition of DNA repair pathways to sensitize glioma cells to radiotherapy or chemotherapy is a well-established and actively investigated strategy. This includes targeting PARP to impair BER, inhibiting DNA-PK or ATM to disrupt NHEJ and HR, respectively, and using ATR or checkpoint kinase 1 inhibitors to abrogate the DNA damage checkpoint response [5]. Combining these inhibitors with TMZ or radiation has produced synergistic effects in preclinical models. For instance, the brain-penetrant compound MTX-241F, which inhibits both EGFR and DNA-PK, exhibits radiosensitizing activity in diffuse intrinsic pontine glioma models [56]. Additionally, natural compounds such as kaempferol inhibit NHEJ repair by stabilizing Ku80 at double-strand break sites, thereby inducing DNA damage accumulation and suppressing glioma growth [58]. These strategies aim to disrupt the tumor’s defensive mechanisms, pushing cells toward lethal levels of genomic instability.

### 7.3. Novel Drug Delivery Systems and Combination Therapies

Overcoming the blood–brain barrier and intrinsic resistance of glioma stem cells requires innovative drug delivery and combination approaches. Nanoparticle-based systems and engineered exosomes are being developed to improve targeted delivery of chemotherapeutic agents and molecular inhibitors to glioma tissues [59]. For example, tumor-derived reassembly exosomes co-loaded with TMZ and dihydrotanshinone have been designed to enhance blood–brain barrier (BBB) penetration, target homologous glioma cells, and reverse TMZ resistance [60]. Combination therapies are also being rationally designed to attack multiple vulnerabilities simultaneously. These include pairing epigenetic modulators such as panobinostat with proteasome inhibitors, which can induce synthetic lethality in resistant cells by creating a dependency on NAD^+^ biosynthesis pathway [61]. Similarly, combining TMZ with ferroptosis-inducing agents, a non-apoptotic cell death pathway, is under investigation as a strategy to bypass classical apoptosis-based resistance mechanism [62]. These multifaceted strategies represent the next frontier in combating this resilient disease.

### 7.4. Exploration of ARGLU1-Based Combination Therapies

Given its role in stimulating DNA damage repair in non-glioma cells, *ARGLU1* represents a candidate target that warrants further investigation for combination strategies with genotoxic chemotherapies or radiotherapy. The roles of *ARGLU1* in DNA damage repair and transcriptional regulation have been established in non-glioma cell lines [7], providing a rationale for exploring its potential involvement in glioma. Overexpression of *ARGLU1* increases cancer cell resistance to genotoxic drugs, suggesting that its inhibition could sensitize tumors to standard DNA-damaging agents [7]. This is directly supported by its inclusion in a gene signature that predicts oxaliplatin resistance in colorectal cancer [63]. Combination approaches could involve pairing an *ARGLU1*-targeted agent with platinum-based chemotherapies to overcome intrinsic or acquired resistance. Furthermore, its interaction with viral proteins such as adenovirus *E1A*, which modulates the DNA damage response [13]. This interaction points to potential synthetic lethal interactions that may be exploited alongside other pathway inhibitors. A summary of the proposed molecular mechanisms and therapeutic implications of *ARGLU1* for glioma is shown in Table 1.

### 7.5. Prospects for ARGLU1-Based Precision Medicine Applications

**Figure 4:**
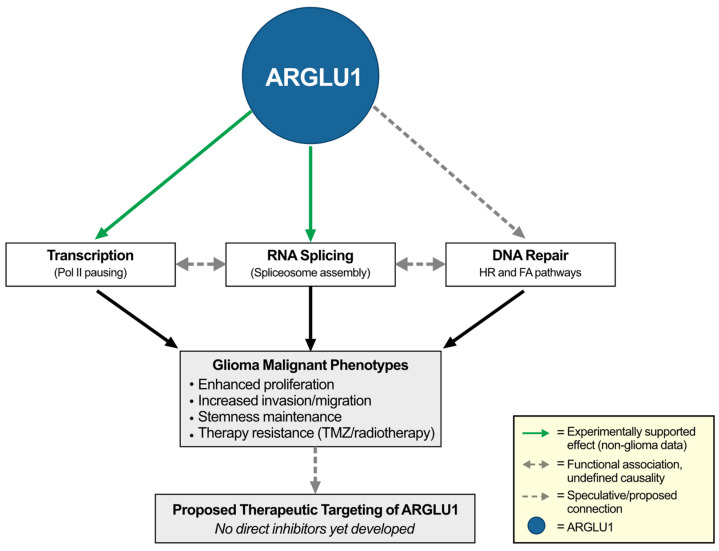
Proposed model of ARGLU1 linking transcription, splicing, and DNA repair in glioma. ARGLU1 directly regulates transcription (via promoter-proximal Pol II pausing) and RNA splicing (solid green arrows), whereas its involvement in DNA repair remains largely phenotypic and emerging (dashed gray arrow). Horizontal bidirectional dashed gray arrows indicate potential regulatory crosstalk among the three functional modules, which remains an open area of investigation in the glioma context. These three processes drive glioma malignant phenotypes, including proliferation, invasion, stemness, and therapy resistance. Therapeutic targeting of ARGLU1 remains speculative (dashed gray arrow). Solid green arrows: experimentally supported effect (non-glioma data). Dashed gray bidirectional arrows: functional association, undefined causality. Dashed gray arrow: speculative/proposed connection. Blue: ARGLU1. Abbreviations: FA, Fanconi anemia; HR, homologous recombination; Pol II, RNA polymerase II; TMZ, temozolomide.

*ARGLU1* is part of a three-gene signature (ORGSig) that predicts oxaliplatin resistance in colorectal cancer, suggesting its potential as a biomarker [63]. However, this finding requires validation in glioma-specific cohorts. Future studies could test whether ARGLU1 expression correlates with response to TMZ, radiotherapy, or PARP inhibitors in glioma patients. If validated, ARGLU1 might help guide treatment decisions, but such applications remain hypothetical (Figure 4).

## 8. Discussion and Future Directions

Based mainly on non-glioma cell line data, ARGLU1 may function as a nuclear protein that could integrate RNA splicing, transcriptional pausing, and DNA damage repair. Whether this occurs in glioma remains unknown. Its ability to simultaneously affect spliceosome assembly, promoter-proximal Pol II pausing, and homologous recombination places this factor at a critical intersection of post-transcriptional regulation and genome stability maintenance. Based on its functions in non-glioma cells (alternative splicing and DNA repair) [7], ARGLU1 may contribute to the resistance of glioma cells to temozolomide and radiotherapy, adding to a major challenge in neuro-oncology.

Consistent with the hypothesis-driven framing in the Introduction, this Discussion focuses on three tasks: (1) summarizing what is experimentally known about ARGLU1, (2) identifying where the evidence is indirect or missing, and (3) proposing testable hypotheses for future work.

The molecular understanding of ARGLU1 has advanced considerably over the past decade. Initial studies identified ARGLU1 as a transcriptional coactivator and splicing regulator important for stress hormone signaling and development [11]. Subsequent work revealed its ability to enhance promoter-proximal RNA polymerase II pausing through interaction with JMJD6, a protein facilitates transcriptional elongation by regulating BRD4 activity [7]. Additional independent studies have reinforced the functional significance of JMJD6 in glioma, demonstrating that its overexpression promotes aggressive tumor phenotypes and correlates with unfavorable patient prognosis, largely due to its role in maintaining oncogenic transcriptional programs [65]. This mechanistic link between ARGLU1 and JMJD6 thus connects transcriptional pausing control to alternative splicing decisions [7]. Recent studies have also shown that ARGLU1 facilitates DNA damage repair, with elevated expression increasing resistance to genotoxic stress and knockdown suppressing cell proliferation [7]. Together, these findings establish ARGLU1 as a versatile regulator operating at the interface of transcription, RNA processing, and DNA repair.

As mentioned in the Introduction, several important limitations must be considered when interpreting the current literature on ARGLU1. Firstly, nearly all functional studies of ARGLU1 have been performed in non-glioma cell lines, including HEK293, U2OS, and various breast or gastric cancer models [7,10,12]. Whether these findings are applicable to glioma cells, particularly glioma stem cells, which exhibit distinct splicing and repair dependencies, remains largely unexplored. Secondly, the proposed direct interaction between ARGLU1 and homologous recombination proteins such as BRCA2 and RAD51 currently relies on protein interaction network predictions and indirect functional assays. Definitive biochemical evidence, including co-immunoprecipitation from native cellular contexts or purified protein binding studies, has yet to be reported. Thirdly, although ARGLU1 is classified as a splicing regulator, high-throughput approaches such as CLIP-seq or RNA-seq following ARGLU1 depletion have not been conducted to identify its direct RNA targets. Consequently, the causal relationship between ARGLU1-mediated Pol II pausing and specific alternative splicing has not been experimentally established.

To preliminarily address the current knowledge gap regarding *ARGLU1* expression in glioma, we analyzed datasets from The Cancer Genome Atlas (TCGA), specifically the TCGA_GBM and TCGA_LGG cohorts, using the GEPIA2 platform (http://gepia2.cancer-pku.cn) [66]. Differential expression analysis using the LIMMA (Linear Models for Microarray Data) method revealed that ARGLU1 was significantly downregulated in glioblastoma (GBM) compared with normal brain tissue (log_2_FC = −2.204, *q* < 0.001), whereas no significant difference was detected in low-grade glioma (LGG) (*q* > 0.05). The expression levels of ARGLU1 in glioblastoma (GBM, *n* = 163) and low-grade glioma (LGG, *n* = 518) compared with normal brain tissue (*n* = 207) are shown in Supplementary Figure S1. These observations suggest that *ARGLU1* downregulation may be specific to high-grade gliomas, contrasting with its proposed oncogenic roles in other cancer types and raising the possibility of context-dependent functions. However, given that all functional studies of ARGLU1 have been conducted in non-glioma cell lines, these findings require experimental validation in glioma-specific models. A comprehensive multi-cohort analysis will be conducted in a follow-up study.

For clarity, the current evidence for *ARGLU1* functions can be categorized into three levels: (1) experimentally validated findings: ARGLU1 enhances promoter-proximal Pol II pausing via JMJD6-BRD4 interaction and promotes DNA damage repair in non-glioma cell lines [7]; (2) indirectly supported findings: ARGLU1 may influence alternative splicing decisions, but direct RNA targets remain unidentified [7,11,64]; (3) hypothetical roles: ARGLU1 interaction with BRCA2/RAD51 lacks direct biochemical evidence, and its prognostic value in glioma also remains unexplored [7].

How does ARGLU1 differ from other splicing regulators? PTBP1, METTL3, and SNRPD2 act directly on RNA or the spliceosome. PTBP1 binds pyrimidine tracts, METTL3 deposits m^6^A, and SNRPD2 is a core spliceosome component. *ARGLU1* works differently. It does not bind RNA. Instead, it controls Pol II pausing via the JMJD6-BRD4 axis, and pausing then influences splicing. Thus, *ARGLU1* is a transcription-coupled splicing regulator, not a classical splicing factor. These findings suggest that *ARGLU1* may cooperate with classical splicing factors, rather than replacing them, and its splicing effects are linked to its roles in transcription and DNA repair.

The function of *ARGLU1* should be interpreted within the broader context of splicing dysregulation in glioma. Multiple splicing factors, including PTBP1, METTL3, and SNRPD2, have been shown to promote oncogenic splicing programs and are associated with unfavorable prognosis [22]. Unlike these dedicated splicing regulators, *ARGLU1* possesses a unique “triple-function” property that encompasses transcriptional pausing, splicing control, and DNA repair coordination. This multifunctionality may explain why *ARGLU1* appears essential for cancer cell proliferation, whereas its absence leads to growth arrest [7,12]. Similarly, alternative splicing of DNA repair genes has emerged as a mechanism of therapy resistance [16,48]. *ARGLU1* may exert its effects on treatment resistance through both direct repair functions and indirect modulation of repair gene splicing. Untangling these interconnected mechanisms is a priority for future investigation.

Splicing dysregulation and enhanced DNA repair jointly drive therapy resistance, and both have been exploited for therapeutic intervention. Pharmacological targeting of spliceosome components or splicing factors has shown preclinical promise, whereas splice-switching oligonucleotides against *BCLX* improve radiosensitivity in GBM models [22,30]. PARP inhibitors similarly display synthetic lethality in mismatch repair-deficient GBM, supporting their combination with temozolomide [42]. *ARGLU1* sits at the nexus of these two resistance mechanisms, making it an attractive but challenging therapeutic target. Its intrinsically disordered nature complicates conventional inhibition but may enable alternative strategies such as PROTACs, and its inclusion in a colorectal cancer resistance signature suggests biomarker potential [63].

Several important questions have yet to be addressed. (1) Beyond our preliminary TCGA-based observation, the expression patterns of *ARGLU1* across glioma grades and subtypes, as well as its prognostic significance, remain to be systematically defined through mining of additional cohorts and validation using tissue microarrays. (2) Its splicing targets in glioma stem cells remain unknown; CLIP-seq, RNA-seq after *ARGLU1* perturbation, and patient-derived models are needed. (3) Direct biochemical evidence for *ARGLU1*-*BRCA2*/*RAD51* binding is absent; co-IP, pull-down, and *in vitro* reconstitution are required. (4) Most data come from non-glioma systems, making orthotopic and patient-derived xenograft validation a priority. (5) The functional coupling between *ARGLU1*’s splicing and repair activities is unclear; structure-function studies could address this. (6) The *JMJD6*-*ARGLU1* axis deserves dedicated investigation in glioma, as JMJD6 promotes progression [65]; whether they modulate each other and affect therapy response remains unknown. (7) ARGLU1’s therapeutic potential must be evaluated via specific inhibitors or degraders, alone or combined with TMZ, radiotherapy, or PARP inhibitors.

In summary, this review makes three contributions. Firstly, it provides the first systematic synthesis of the proposed triple functions of *ARGLU1* in glioma. Secondly, it clearly distinguishes experimentally validated findings from indirect evidence and hypothetical roles. Thirdly, it defines *ARGLU1* as a transcription-coupled splicing regulator, distinguishing it from classical splicing factors.

## 9. Conclusions

ARGLU1 has evolved from a protein implicated in splicing to a regulator that may bridge transcription, RNA processing, and genome stability. However, this model needs validation in glioma-specific models. Its potential roles in glioma pathogenesis and treatment resistance highlight the importance of coordinated splicing and repair pathways in cancer. Substantial gaps still exist, especially regarding *ARGLU1*’s direct molecular targets, its prognostic significance in glioma, and its suitability for pharmacological targeting. The foundation for mechanistic and translational studies of *ARGLU1* has been established. *ARGLU1* not only enriches our understanding of glioma biology but also points toward therapeutic opportunities. Addressing these opportunities will require integrated multidisciplinary efforts spanning functional genomics, chemical biology, and clinical investigation.

## Figures and Tables

**Table 1:** Summary of Proposed Molecular Mechanisms and Therapeutic Implications of *ARGLU1* for Glioma.

Category	Aspect	Mechanism	Therapeutic Strategy	Key References	Evidence Level
Oncogenic Function	Pro-tumorigenic roles	*ARGLU1* acts as an oncogene promoting cell proliferation and survival. Its overexpression correlates with malignant progression.	Validates *ARGLU1* as a rational therapeutic target.	[7,10,12,18]	Experimentally validated (non-glioma data)
Oncogenic Function	Signaling pathway modulation	*ARGLU1* may regulate PI3K/AKT signaling and influence MAPK/ERK pathways, contributing to uncontrolled cell growth and apoptosis evasion.	High *ARGLU1* expression could predict sensitivity or resistance to PI3K/AKT inhibitors.	[7,12]	Indirectly supported
RNA Splicing Regulation	Spliceosome modulation	ARGLU1 modulates spliceosome assembly and promoter-proximal Pol II pausing via JMJD6-BRD4 interaction, influencing alternative splicing decisions.	Targeting *ARGLU1* could disrupt oncogenic splicing programs; synthetic lethality with spliceosome inhibitors is plausible.	[7,22,64]	Experimentally validated (non-glioma data)
RNA Splicing Regulation	Targetable AS events	*ARGLU1* regulates specific AS events generating actionable vulnerabilities: *OY-TES-1* V5a (proliferation/invasion), *RCAN1-4* (TCR-T neoepitope), *BCLX* (Bcl-xL, apoptosis resistance), *NRCAM* (Δex5Δex19, surface proteoform).	Therapeutic strategies include SSOs (*BCLX*), TCR-T cell therapy (*RCAN1-4*), and monoclonal antibodies (*NRCAM*).	[26,27,28,29,30,31,32]	Mixed: only *BCLX* validated; others speculative
RNA Splicing Regulation	Stem cell maintenance	*ARGLU1* may sustain stem cell phenotype through alternative splicing of stemness-associated genes (e.g., *CERS5*, *MPZL1*), driving tumor progression and therapy resistance.	Targeting *ARGLU1* could eliminate stem cells and overcome resistance.	[7,22,39]	Indirectly supported
DNA Repair Coordination	Homologous recombination repair	ARGLU1 may synergize with HR pathway via interaction with BRCA2/RAD51, enhancing DSB repair efficiency and promoting resistance to DNA-damaging agents.	*ARGLU1* inhibition could sensitize tumor cells to TMZ and radiotherapy.	[7,13,17]	Indirectly supported (needs glioma-specific testing)
DNA Repair Coordination	PARP inhibitor sensitivity	*ARGLU1* overexpression may confer PARPi resistance by compensating for HR defects; *ARGLU1* knockdown sensitizes cells to PARP inhibitors.	*ARGLU1* expression status could predict PARPi efficacy in HR-deficient tumors.	[7,13,42,47]	Hypothetical (requires glioma validation)
Precision Medicine	Prognostic biomarker	ARGLU1 expression levels correlate with tumor grade and patient survival (preliminary evidence from colorectal cancer [63]), but glioma-specific validation is needed.	Patient stratification based on ARGLU1 expression could optimize therapeutic precision.	[63]	Hypothetical (needs glioma-specific validation)
Precision Medicine	Combination therapy	*ARGLU1* inhibition sensitizes cancer cells to TMZ, radiotherapy, and PARP inhibitors; viral *E1A* interaction suggests synthetic lethal vulnerabilities.	Rational combinations: *ARGLU1*-targeted agents with TMZ, radiotherapy, or PARP inhibitors.	[7,13,42]	Indirectly supported / hypothetical

Abbreviations: AS, alternative splicing; *BCLX*, BCL2-like gene X; *BRCA2*, breast cancer type 2 susceptibility protein; BRD4, bromodomain-containing protein 4; *CERS5*, ceramide synthase 5; DSB, double-strand break; *E1A*, adenovirus early region 1A; HR, homologous recombination; JMJD6, jumonji domain-containing protein 6; MAPK, mitogen-activated protein kinase; *MPZL1*, myelin protein zero-like 1; *NRCAM*, neuronal cell adhesion molecule; PARP, poly(ADP-ribose) polymerase; PI3K, phosphoinositide 3-kinase; Pol II, RNA polymerase II; *RCAN1-4*, regulator of calcineurin 1 isoform 4; SSO, splice-switching oligonucleotide; TCR-T, T cell receptor-engineered T cell; TMZ, temozolomide.

## Data Availability

No new data were created or analyzed in this study. Data sharing is not applicable to this article.

## Supplementary Materials

The following supporting information can be downloaded at: https://www.mdpi.com/article/10.3390/cells15121124/s1, Figure S1: ARGLU1 expression in glioma from TCGA data.

## Abbreviations

The following abbreviations are used in this manuscript:

ARGLU1	Arginine and Glutamate Rich 1
AS	Alternative splicing
ATR	Ataxia telangiectasia and Rad3-related protein
BBB	Blood–brain barrier
BCLX	Bcl-extra
BER	Base excision repair
BRCA2	Breast cancer type 2 susceptibility protein
BRD4	Bromodomain-containing protein 4
C/EBPβ	CCAAT/enhancer-binding protein beta
circRNA	Circular RNA
CLIP-seq	Crosslinking immunoprecipitation coupled with high-throughput sequencing
CTNNB1	Catenin beta 1
DDR	DNA damage response
DNA-PK	DNA-dependent protein kinase
DSB	Double-strand break
FA	Fanconi anemia
GBM	Glioblastoma
GSC	Glioma stem cell
HLA-A24	Human leukocyte antigen A24
HNRNPH1	Heterogeneous nuclear ribonucleoprotein H1
HR	Homologous recombination
IDH	Isocitrate dehydrogenase
ITSN1	Intersectin 1
JMJD6	Jumonji domain-containing protein 6
LIMMA	Linear Models for Microarray Data
LGG	Low-grade glioma
LSM2	U6 snRNA-associated Sm-like protein 2
MBNL1	Muscleblind-like splicing regulator 1
MED1	Mediator complex subunit 1
METTL3	Methyltransferase-like 3
MGMT	O^6^-methylguanine-DNA methyltransferase
MMR	Mismatch repair
MS4A7	Membrane spanning 4-domains A7
MUTYH	MutY DNA glycosylase
NELF	Negative elongation factor
NHEJ	Non-homologous end joining
NRCAM	Neuronal cell adhesion molecule
PARP	Poly(ADP-ribose) polymerase
PARPi	PARP inhibitor
pHGG	Pediatric high-grade glioma
Pol II	RNA polymerase II
PROTAC	Proteolysis-targeting chimera
PSI	Percent spliced-in
PTBP1	Polypyrimidine tract-binding protein 1
PTBP2	Polypyrimidine tract-binding protein 2
RAD51	RAD51 recombinase
RCAN1	Regulator of calcineurin 1
RBP	RNA-binding protein
RNA-seq	RNA sequencing
SNRPD2	Small nuclear ribonucleoprotein D2 polypeptide
SP1	Specificity protein 1
SSO	Splice-switching oligonucleotide
TCF12	Transcription factor 12
TCGA	The Cancer Genome Atlas
TCR	T cell receptor
TCR-T	T cell receptor-engineered T cell
TMZ	Temozolomide
YAP	Yes-associated protein
YY1	Yin Yang 1
