# Supplementary figures and images for: ARGLU1 in Glioma: A Novel Potential Regulator of Splicing, DNA Repair, and Therapeutic Resistance
Source: Cells. 2026 Jun 22;15(12):1124. doi: 10.3390/cells15121124 (PMC13297544; doi:10.3390/cells15121124)

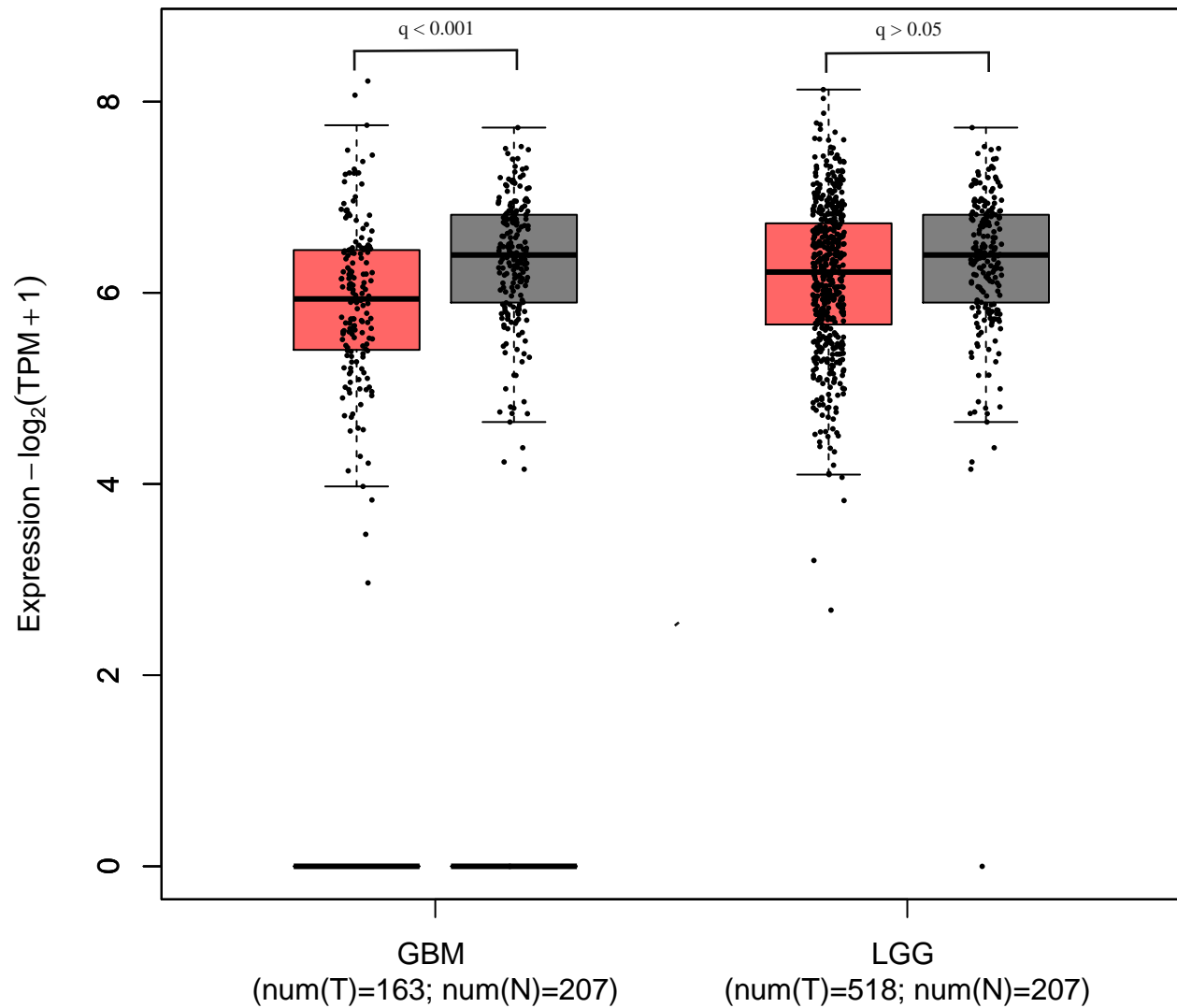

Supplement: Supplementary file 1 [file cells-15-01124-s001.zip › cells-4319013-supplementary.pdf]
